# Redesigning the Barefoot Doctor Model for Rural Syria: A Telemedicine-Enhanced Policy Framework

**DOI:** 10.1055/s-0046-1827455

**Published:** 2026-08-12

**Authors:** Mohannad M. Alarqan

**Affiliations:** 1 Independent Researcher, Healthcare Economics, Amman, Jordan

**Keywords:** health workforce, rural health services, telemedicine, primary health care, Syria

## Abstract

Rural Syria faces a workforce problem that is not only numerical but allocative: physician-led recovery is slow, urban-biased, and poorly matched to dispersed communities living under insecurity, damaged infrastructure, and fragmented governance. This correspondence argues that the historical barefoot doctor model should not be revived literally, but redesigned as a supervised mid-level primary care cadre embedded within telemedicine-supported governance. Drawing on evidence from Syria, humanitarian and post-conflict community health worker literature, telemedicine experience in conflict-affected Eastern Mediterranean settings, and World Health Organization guidance, this article proposes a policy framework with four linked functions: focused training, needs-based deployment, remote clinical supervision, and structured referral and learning. The aim is to expand rural access while protecting clinical safety and reducing inefficient use of scarce physician time.

More than a decade of conflict has left Syria with a damaged, fragmented, and territorially uneven health system. Attacks on health facilities, workforce losses, interrupted training pipelines, and unstable governance have narrowed the practical options for rural service recovery. 1 2 Conventional physician-centered reconstruction remains important, but it is slow, capital-intensive, and likely to remain concentrated in better-served areas. In rural Syria, the binding constraint is not simply too few clinicians. It is also the inefficient allocation of scarce physician time across routine primary care, referral management, supervision, and complex clinical tasks. From a healthcare economics perspective, this creates a persistent access gap with high opportunity cost. 3 4

The historical barefoot doctor model remains analytically useful because it captures an old but still relevant policy intuition: health systems facing severe workforce scarcity may need a lower-cost, locally embedded frontline cadre. However, the original model should not be copied. Evidence from humanitarian and post-conflict settings suggests that community and mid-level providers can improve access, continuity, and early case detection when they are selected locally, trained for a defined package of care, supervised regularly, and integrated into wider service delivery structures. 3 4 5 Yet the same literature warns that weakly governed delegation can shift clinical risk to patients and to poorly supported workers. 4 5 For Syria, the question is therefore not whether task-shifting is conceptually acceptable, but how it can be governed in a way that protects safety, accountability, and value.

This correspondence proposes a telemedicine-enhanced redesign with four linked policy functions. First, a mid-level rural primary care cadre should be trained through a focused, competency-based program with a restricted scope of practice covering common infections, maternal and child health support, chronic disease follow-up, basic triage, and referral initiation. Second, deployment should be guided by rural access deficits rather than by administrative habit, using simple indicators such as distance to functioning facilities, travel difficulty, displacement pressure, and local service burden. Third, telemedicine should be treated not as an isolated digital add-on but as clinical governance infrastructure. Low-cost remote consultation can connect frontline providers to physician supervisors for case discussion, protocol support, referral validation, and continuing education. 6 7 8 Fourth, routine digital reporting should feed simple system learning by tracking referral patterns, common presentations, safety events, and training needs.

The economic value of this model lies in better task allocation rather than in technology alone. If routine problems are managed locally within a governed scope of practice, scarce physician time can be redirected toward complex diagnosis, supervisory review, protocol design, and hospital-level care. This may reduce unnecessary travel, avoid some low-value referrals, and improve horizontal equity in geographically underserved communities. 6 7 Any implementation should remain cautious. A pilot would need clear stewardship, explicit referral rules, digital privacy safeguards, financing for supervision and connectivity, and prospective monitoring of patient safety, service use, and physician time allocation. In short, redesigning the barefoot doctor idea for rural Syria is defensible only if it is redesigned as a governed health workforce model rather than revived as a low-cost substitute for formal medicine.
